# Performance Evaluation and Exponential Prediction Models for LDPE-Substituted Concrete and Mortar

**DOI:** 10.3390/polym18101263

**Published:** 2026-05-21

**Authors:** Omer Fatih Sancak, Muhammet Zeki Ozyurt, Gamze Demirtas, Sarah S. M. A. Sayed

**Affiliations:** 1 Department of Construction Technology, Dogus University, Istanbul 34722, Turkey; osancak@dogus.edu.tr; 2 Department of Civil Engineering, Sakarya University, Sakarya 54050, Turkey; demirtas@sakarya.edu.tr; 3 Institute of Natural Sciences, Sakarya University, Sakarya 54050, Turkey; sarah.sayed@ogr.sakarya.edu.tr

**Keywords:** LDPE, exponential model, concrete, mortar, sustainability

## Abstract

The increasing use of low-density polyethylene (LDPE) has prompted growing interest in its application as a replacement for conventional aggregates in concrete. This study investigated the effects of replacing sand with 10%, 20%, and 30% LDPE granules in concrete. Compressive strength, splitting tensile strength, flexural strength, modulus of elasticity, slump, and density tests were performed. The results showed a gradual decrease in compressive strength (from 26.91 MPa in the reference mix to 16.56 MPa with 30% LDPE), tensile strength (from 2.46 MPa to 1.84 MPa), and flexural strength (from 3.37 MPa to 2.59 MPa). Decreases were also observed in modulus of elasticity, slump, and density values. However, LDPE-substituted concretes increased their axial and lateral strain capacities, showing improvement in ductility and deformation ability. Experimental results demonstrated a delicate balance between mechanical strength and sustainability benefits. It was demonstrated that low rates of LDPE substitution could balance performance with environmental advantages. The experimental results presented in this study were combined with previous research to create a dataset. Based on this dataset, exponential models predicting the properties of LDPE-substituted concrete and mortar were proposed. The proposed exponential models outperformed existing linear models in prediction accuracy, yielding coefficient of determination (R^2^) values up to 0.981 and significantly reduced mean absolute percentage error (MAPE) values, ranging from 1% to 17% depending on the dataset.

## 1. Introduction

The accumulation of non-biodegradable plastic waste has become one of the most significant environmental problems of our time. Global plastic production has increased dramatically over the last fifty years, and polyethylene (PE) has become one of the most common types of plastic waste [1,2,3,4,5]. Traditional disposal methods such as landfilling or incineration not only lead to the use of valuable land but also increase environmental pollution and greenhouse gas emissions [6]. Therefore, researchers are increasingly investigating the use of waste plastics in construction materials to both reduce the waste management problem and develop new materials.

Concrete, one of the most commonly used materials in the construction sector, offers a significant opportunity for the recovery of plastic waste. Using plastics in concrete can both reduce the amount of waste going to landfills and decrease the need for natural aggregates. Therefore, plastic-modified concrete is seen as a viable solution compatible with the circular economy approach and economically advantageous in resource-scarce regions [7,8,9,10,11]. Studies have shown that replacing natural aggregates with polyethylene types such as high-density polyethylene (HDPE) and low-density polyethylene (LDPE) can reduce the thermal conductivity and energy consumption of building elements [6,12]. Furthermore, the use of LDPE instead of fine aggregate has been investigated, and X-ray diffraction analyses have shown that this material has a partially crystalline structure and can be compatible with the cement matrix [13]. In studies where LDPE pellets were used in permeable concrete, it was observed that abrasion resistance increased, but compressive strength generally decreased [14]. In addition, it has been reported that recycled plastics can be used as aggregates, fibers, or additives in concrete and asphalt mixtures and can provide benefits such as reduced density, increased crack resistance, and improved durability under appropriate conditions [15,16].

In addition to concrete applications, innovative methods have emerged in developing countries. One of these methods is melting waste LDPE bags and mixing them with sand. Durable pavement blocks are produced from this mixture. These materials are called LDPE binder sand composites. And they can achieve compressive strengths close to concrete. This method offers both environmental and economic benefits in regions where recycling infrastructure is insufficient [17]. In addition, LDPE has been used in cementless pavement blocks, lightweight concrete, and self-compacting concrete. In these applications, the use of LDPE generally contributes to reduced density, increased flexural strength, and improved thermal properties [18,19]. Furthermore, it has been determined that PE-containing mixtures can exhibit sufficient mechanical performance in some non-structural elements. The use of LDPE in concrete is being investigated, particularly in terms of its potential to reduce thermal conductivity and increase the ductility of the material [20,21].

Studies on polymer-modified asphalt concretes are also noteworthy. Studies have shown that mixing HDPE and LDPE-containing aggregates with dry or wet mixing methods can improve rutting resistance, Marshall stability, stiffness, and durability [22,23,24,25,26]. Similarly, studies where recycled concrete aggregates were mixed with polyethylene granules yielded stiffness and modulus of elasticity values that could be suitable for pavement base and substrate applications [27]. Other studies also show that replacing some of the natural aggregates in asphalt mixes with LDPE or HDPE aggregates can improve rutting resistance, modulus of elasticity, and modulus of dynamics [28].

While there are advantages to using LDPE in some structural materials, there are also some disadvantages. Substituting LDPE in concrete generally leads to a decrease in compressive strength. This can be attributed to the weak bond between LDPE particles and cement [29]. However, some studies show that LDPE is feasible when used in low proportions. Substitution rates between 5% and 20% are generally recommended to achieve a suitable balance between the mechanical performance of the building material and the environmental benefit [30,31]. Furthermore, studies highlight that recycled LDPE can offer advantages in terms of thermal insulation, durability, and sustainability. However, attention should be paid to the percentage of substitution to minimize strength losses.

Prior investigations [32,33] into the utilization of LDPE as a replacement material in construction have introduced predictive models aimed at estimating mechanical performance. Among these contributions, one study developed equations suggesting that the extent of LDPE substitution exerts an influence on both compressive and tensile strengths through linear relationships. The equation for compressive strength suggested in [32] is presented in Equation (1), whereas the corresponding tensile strength relationship is provided in Equation (2). In these equations, ldpe% represents the percentage of LDPE incorporated as a replacement, $f_{tldpe}^{\prime }$ represents the tensile strength of those specimens, and $f_{cldpe}^{\prime }$ represents the compressive strength of specimens containing LDPE.

(1) $$f_{cldpe}^{\prime }=0.9738-0.0078(ldpe\%)$$

(2) $$f_{tldpe}^{\prime }=1.0007-0.0056(ldpe\%)$$

In this research, LDPE was incorporated into concrete as a partial substitute for river sand, with replacement levels of 10%, 20%, and 30% by volume. This approach was adopted to support the preservation of natural resources while simultaneously enhancing the recycling of LDPE. In addition to the experimental program, results from similar studies on concrete and mortar reported in the literature were compiled. And the relationships among the experimental results were examined. Based on this analysis, exponential models were proposed for LDPE-substituted concrete and mortar instead of the previously suggested linear models. Despite the growing number of studies on LDPE-modified concrete, limited research has focused on developing reliable predictive models for estimating mechanical properties based on LDPE content. Therefore, this study aims to address this gap by proposing and validating exponential prediction models based on an extensive experimental database.

## 2. Materials and Methods

Grain size distributions of the aggregates were determined by performing individual sieve analyses on the coarse and fine aggregate (crushed limestone and river sand), following the methodology outlined in EN 933-1 [34]. Table 1 presents the sieve analysis results for crushed limestone.

Table 2 presents the sieve analysis results for river sand.

**Figure 1:**
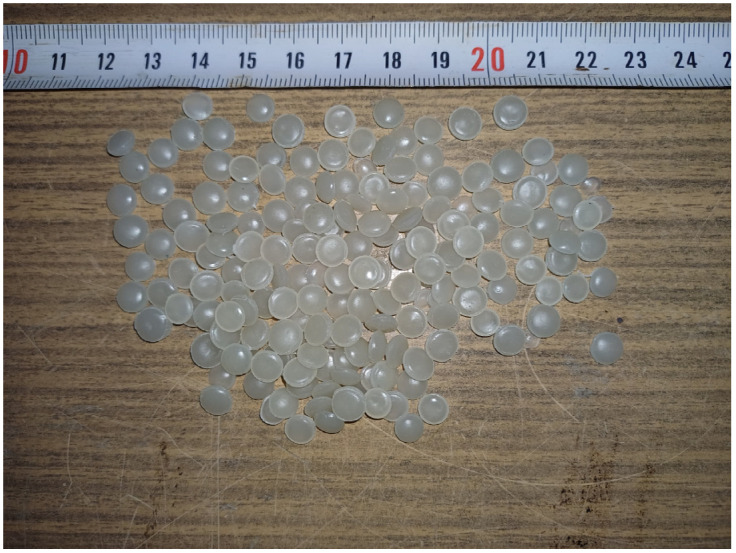
LDPE granules.

CEM I 42.5 R type Portland cement was used in the concrete mixes. This cement conforms to the properties defined in EN 197-1 [35] standard. The LDPE granules employed in this investigation were sourced from a recycling plant located in Türkiye. A visual representation of LDPE granules is given in Figure 1. The properties of LDPE, coarse aggregate, and fine aggregate are summarized in Table 3.

Coarse and fine aggregate, cement, LDPE, and water were added to a mechanical mixer and the machine was started. This ensured proper distribution, and concrete samples were prepared. To maintain consistency throughout the experiment, the ratios of coarse aggregate, cement, and water were kept constant. The water-to-cement ratio was chosen as 0.5. 10%, 20%, and 30% by volume of river sand were replaced with LDPE. The reference mixture (0% LDPE) was designed to achieve a target strength consistent with the C25/30 concrete class, providing a standard structural baseline for the evaluation of LDPE-modified samples.

The mix proportions of all concrete specimens are presented in Table 4. The quantities of each material were kept constant except for the fine aggregate, which was partially replaced by LDPE at specified ratios. A coding method was used to distinguish the samples during the test. The details of the sample name coding are given in Table 4.

Fresh concrete was subjected to a slump test and evaluated for workability. Specimens of cylindrical geometry (100 mm × 200 mm) were produced for testing both compressive strength and splitting tensile strength. Beams with dimensions of 100 × 100 × 400 mm were prepared for flexural strength testing. Compressive, splitting tensile, and flexural strength specimens were subjected to water curing for 28 days. The curing process was carried out in a water tank at a temperature of 20 ± 2 °C to ensure consistent hydration conditions for all specimens. They were then dried, weighed, and their densities determined.

**Figure 2:**
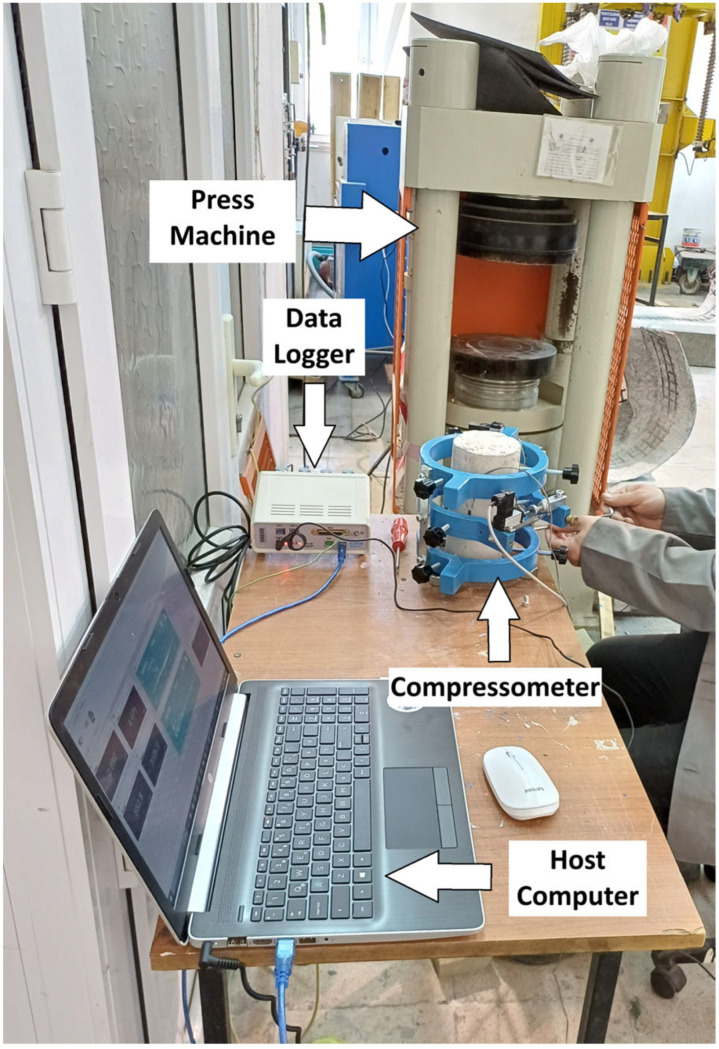
Experimental setup for the compressive strength test.

A compressometer was used for compressive strength evaluation. To ensure the reliability of the results, axial and lateral deformations were monitored using two independent measurement systems: potentiometers connected to a compressometer and strain gauges attached directly to the specimens. The displacement values obtained from these two systems were compared and found to be in close agreement. The reported deformation values were determined by averaging the results obtained from both measurement techniques. All compressive strength tests were conducted under controlled loading conditions, and the loading rate was maintained constant at 0.5 MPa/s to ensure consistency and comparability of results. Stress and strain values were obtained. The experimental setup is shown in Figure 2.

In the splitting tensile strength test, a cage was used to subject cylindrical specimens to tensile force. During the test, the specimens, which were previously placed vertically in the compressive strength test, were this time placed horizontally on the machine. Each specimen was loaded at a constant rate of 0.5 MPa/s. The highest load withstood before fracture was considered the splitting tensile strength.

The flexural performance of the concrete was assessed using prismatic beam specimens subjected to a three-point bending configuration. In this setup, the support span was fixed at 300 mm, and the loading was applied at a constant displacement rate of 5 mm/min.

## 3. Concrete Tests

Specimens of concrete were tested for slump, density, and mechanical properties including compressive, tensile, and flexural strengths. The full dataset is reported in Table 5.

### 3.1. Compressive Strength Test

**Figure 3:**
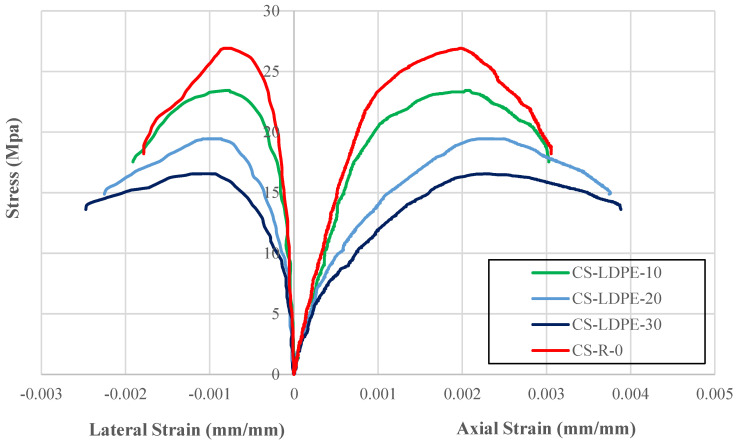
Stress-strain behavior of test specimens.

Figure 3 displays the stress–strain responses generated during the compressive strength testing. The corresponding values of peak compressive strength ($f_{co}^{\prime }$), the axial strain at peak stress ($\varepsilon_{co}$), and the lateral strain recorded at the same stress level ($\varepsilon_{lo}$) are summarized in Table 6. According to the test results, the reference concrete mixture without LDPE exhibited a compressive strength of 26.91 MPa.

A gradual decrease in compressive strength occurred as a result of increasing LDPE substitution for fine aggregate. At a 10% substitution level, the strength decreased by 12.93% and fell to 23.43 MPa. When the substitution rate was 20%, the decrease became more pronounced, reaching 27.73%, and the strength became 19.45 MPa. The most significant decrease occurred at 30% substitution, and the strength decreased by 38.45% to 16.56 MPa. The results indicate that incorporating polymer-based materials leads to a reduction in the concrete’s load-bearing capacity.

Although compressive strength decreased with increasing LDPE substitution percentage, axial and lateral strain values increased. Mixtures containing LDPE showed greater deformation capacity before fracture. Specifically, axial strain increased by 6.25%, 11.60%, and 17.19% at 10%, 20%, and 30% LDPE substitution levels, respectively. Lateral strain increased by 9.82%, 38.12%, and 48.75% at the same substitution rates. These results indicate that LDPE substitution reduces the strength of concrete while simultaneously increasing its ductility.

The reduction in strength may be attributed to the low stiffness and weak interfacial bonding between LDPE particles and the cement matrix. In addition, differences in elastic properties between LDPE and river sand may lead to stress concentrations within the interfacial transition zone. However, the higher strain measurements suggest that LDPE-modified mixtures can undergo greater deformation before fracture. This property may be advantageous in applications where deformation capacity and ductility are prioritized over high compressive strength. This increased deformability can improve energy dissipation and seismic performance, as the material resists brittle failure by enduring larger strains.

### 3.2. Splitting Tensile Strength Test

Increasing the level of LDPE substitution decreased the splitting tensile strength. At 10% substitution, the strength decreased by approximately 8.22% to 2.26 MPa. At 20% substitution, the strength decreased by approximately 18.37% to 2.01 MPa. The most significant decrease occurred at 30% substitution. At 30% substitution, the splitting tensile strength decreased by approximately 25.26% to 1.84 MPa. The results obtained from the splitting tensile strength test are given in Table 7.

### 3.3. Flexural Strength Test

**Figure 4:**
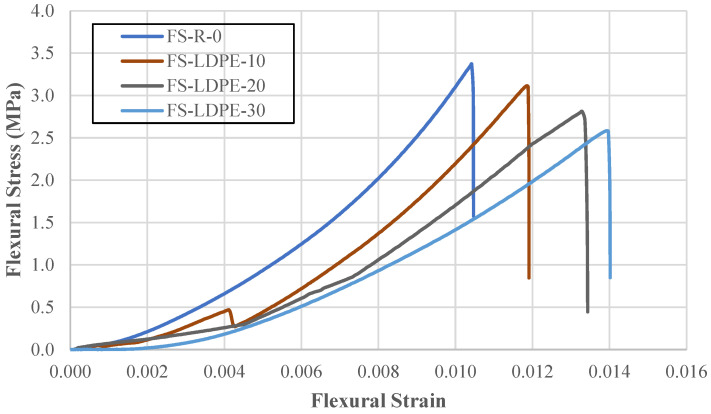
Flexural stress–flexural strain behavior of test specimens.

Flexural strength was determined by means of a three-point bending test. Figure 4 displays the stress–strain responses, and Table 8 summarizes the peak strength and strain values.

A gradual decrease in flexural strength was observed with increasing LDPE substitution for river sand. At 10% substitution, the strength decreased by 7.61% to 3.12 MPa. At 20% substitution, the strength decreased by 16.64% to 2.81 MPa. The most significant decrease occurred at 30% substitution. At 30% substitution, the strength decreased by 23.35% to 2.59 MPa. These results show that LDPE substitution reduces the maximum flexural stress values of the concrete.

Conversely, the maximum strain values increased with higher LDPE content. With 10% substitution, the flexural strain increased by 13.76%. At 20%, the increase was 28.33%. And at 30%, it increased by 33.90%. This shows that despite the decrease in flexural strength, concrete with LDPE substitution has the ability to deform further before fracturing.

### 3.4. Modulus of Elasticity Test

Table 9 presents the modulus of elasticity values determined through compressive strength tests, calculated from the associated stress–strain responses. The modulus of elasticity of concrete without LDPE was calculated as 30,574.17 MPa.

A decrease in the modulus of elasticity was observed with increasing LDPE substitution for river sand. At 10% substitution, the modulus of elasticity decreased by 15.57% to 25,815.23 MPa. At 20% substitution, a modulus of elasticity of 21,027.20 MPa was obtained, a decrease of 31.23%. The most significant decrease occurred at 30% substitution. At 30% substitution, the modulus of elasticity decreased by 34.46% to 20,039.28 MPa.

### 3.5. Slump Test

**Figure 5:**
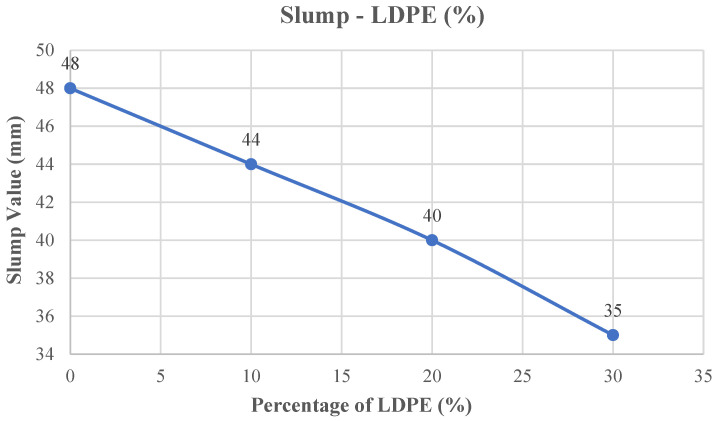
Slump value–LDPE content curve.

The concrete slump test results are shown in Figure 5. The slump value of the mixture without LDPE was measured as 48 mm. The slump values decreased continuously as a result of replacing river sand with LDPE. When 10% of the river sand was substituted with LDPE, the slump decreased by 8.33%, reaching 44 mm. At a 20% replacement level, the slump dropped by 16.67% to 40 mm. The most pronounced reduction was observed at 30% replacement. When 30% was replaced, the slump value decreased by 27.08% to 35 mm.

### 3.6. Density Test

**Figure 6:**
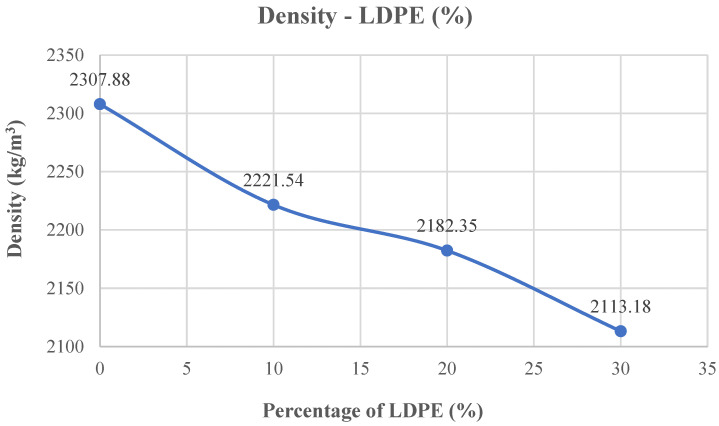
Density value–LDPE content curve.

The density outcomes of the concrete mixtures are illustrated in Figure 6. For the reference mix without LDPE, the measured density was 2307.88 kg/m^3^.

Substituting LDPE for river sand caused a decrease in density. At 10% substitution, the density decreased by 4.02% to 2215.18 kg/m^3^. At 20% substitution, it decreased by 5.73% to 2175.53 kg/m^3^. The most significant decrease occurred at 30% substitution. At 30% substitution, the density decreased by 8.93% to 2101.71 kg/m^3^.

## 4. Assembly of Experimental Results and Development of Models

This section aims to develop models that predict various properties of concrete and mortar containing LDPE. To this end, a comprehensive dataset was compiled. This dataset was created using data from previous studies where LDPE was used as a partial substitute for fine aggregate. Experimental results obtained in this study were also included in this dataset. The experimental data compiled for LDPE-substituted concrete and mortar are summarized in Table 10.

The table compiled in this study is based on a comprehensive literature review conducted under specific boundary conditions. Only studies involving fine aggregate substitution were considered, while those including coarse aggregate substitution were excluded. In addition, only granular LDPE substitution was taken into account, and fiber-based substitutions were not included. The dataset is limited to concrete and mortar samples, excluding self-compacting concrete. Furthermore, only LDPE-substituted mixtures were considered, and no other types of plastic were included. For compressive strength (CS), tensile strength (TS), and flexural strength (FS), only 28-day test results were collected. In cases where cube specimens were used to determine compressive and tensile strengths, equivalent cylinder strengths were calculated by applying a conversion factor of 0.8 and reported separately in the table. The modulus of elasticity (MoE) values are also included. Details regarding sample dimensions are provided; if not explicitly stated, the relevant testing standard is specified instead. Additionally, the table presents the specific gravity, bulk density, and particle size of the LDPE material, as well as the substitution percentage. Concrete density values, water-to-cement (W/C) ratios, and slump test results are also reported in the table.

Using the assembled experimental database, new predictive relationships were established to evaluate the main mechanical and physical characteristics of concrete and mortar containing LDPE. These models were formulated to estimate properties such as modulus of elasticity, compressive strength, flexural strength, and tensile strength. Their reliability was subsequently examined by comparing the predictions with equations previously reported in the literature.

The agreement of model estimates with experimental observations was calculated using the statistical indicators given in Table 11. These statistical indicators are mean absolute percentage error (MAPE), mean absolute bias error (MABE), and the coefficient of determination (R^2^). The R^2^ value indicates the quality of fit of the model with the variance in the experimental data. MABE represents the mean absolute deviation between the estimated and observed values. MAPE expresses this deviation as a percentage [40,41,42,43].

In Table 11, ${exp}_{i}$ denotes individual experimental measurements, whereas $mod_{i}$ corresponds to the respective model predictions. The terms ${\bar{exp}}_{i}$ and ${\bar{mod}}_{i}$ indicate the mean values of the experimental results and model outputs, respectively.

### 4.1. Formulation of the Compressive Strength Prediction Model

The compressive strength values reported in earlier studies are given in Table 11. In order to examine the influence of LDPE incorporation on compressive strength, a compressive strength ratio ($\frac{f_{cldpe}^{\prime }}{f_{c}^{\prime }}$) was determined. This ratio was calculated by dividing the compressive strength of concrete containing LDPE by that of the corresponding reference concrete mixture. In this formulation, ($f_{cldpe}^{\prime }$) is defined as the compressive strength of concrete incorporating LDPE, whereas ($f_{c}^{\prime }$) corresponds to the compressive strength measured for the control specimen.

**Figure 7:**
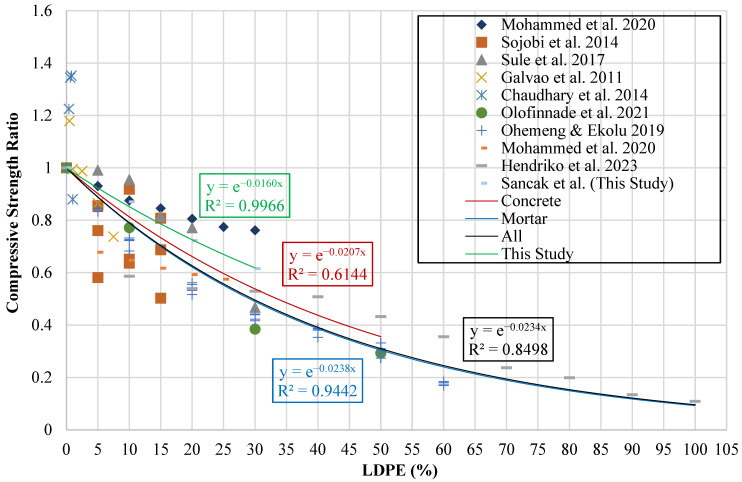
Relationship curve between compressive strength ratio and LDPE content [2,31,32,33,36,37,38,39].

The influence of LDPE replacement is depicted in a graph where the vertical axis represents the compressive strength ratio and the horizontal axis indicates the percentage of LDPE substitution (ldpe%). Ratios exceeding unity signify an enhancement in compressive strength due to LDPE incorporation, whereas values below unity reflect a reduction. The corresponding graphical illustration is provided in Figure 7.

Trendlines were applied to the data to formulate the effect of LDPE on experimental results. These lines were generated to represent the experimental outcomes obtained in the present study, mortar mixtures, concrete mixtures, as well as the complete dataset. Exponential trend lines were found to provide more accurate predictions compared to linear trend lines. The resulting exponential equations are presented in Table 12.

Previous models proposed in the literature for LDPE-modified concrete and mortar, and the models proposed in this study, were evaluated using statistical parameters. The results of this evaluation are presented in Table 13 and Table 14.

**Figure 8:**
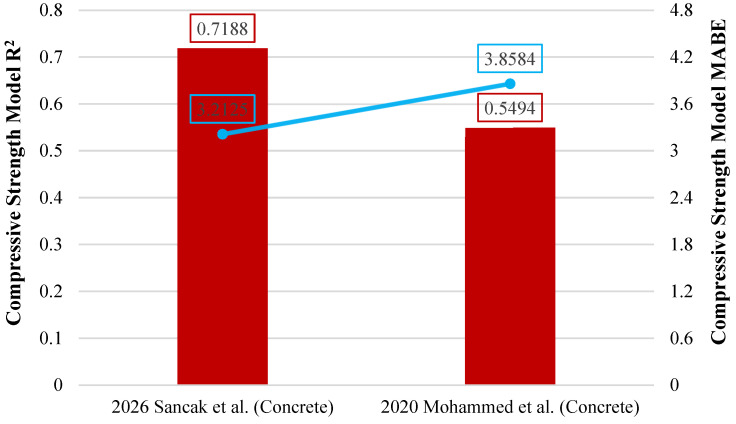
Comparison of compressive strength models for concrete based on R^2^ and MABE [32].

The model proposed by Sancak et al. for estimating the compressive strength of LDPE-substituted concrete demonstrated higher performance than the previously proposed model by Mohammed et al. [32], with an R^2^ value of 0.719. The use of a linear function in the Mohammed et al. model [32] had a negative effect on the R^2^, MABE, and MAPE values. In contrast, the exponential function proposed by Sancak et al. was found to be more compatible with the response of concrete to increasing LDPE ratios. Figure 8 presents a graphical evaluation of the models, displaying R^2^ and MABE metrics.

Table 14 provides a performance comparison of the proposed models for LDPE-substituted mortar. In Table 14, the symbol $\gamma_{ldpe}$ denotes the density of mortar incorporating LDPE, w/c denotes water-to-cement ratio, P denotes LDPE content and T denotes curing age.

**Figure 9:**
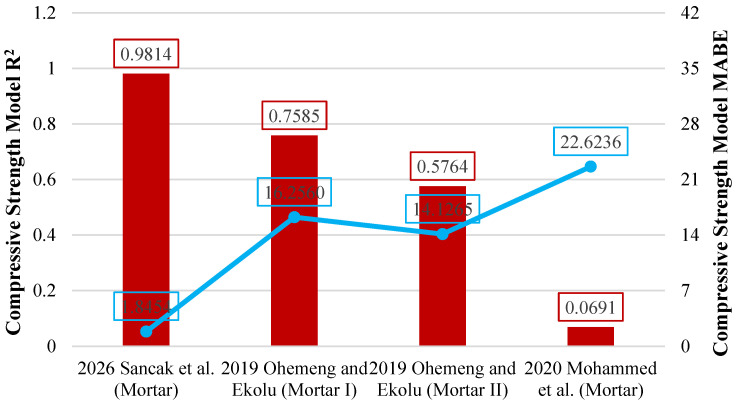
Comparison of compressive strength models for mortar based on R^2^ and MABE [32,33].

Among the evaluated models, the model proposed by Sancak et al. provided the highest prediction accuracy for the compressive strength of LDPE-substituted mortar. This model has an R^2^ value of 0.981. The Ohemeng and Ekolu [33] (Mortar I) model came in second with an R^2^ value of 0.759. In contrast, the model introduced by Muhammed et al. [32] showed a very poor prediction performance. This model has only an R^2^ value of 0.069. Figure 9 presents a graphical comparison of the R^2^ and MABE values of these models.

### 4.2. Formulation of the Tensile Strength Prediction Model

**Figure 10:**
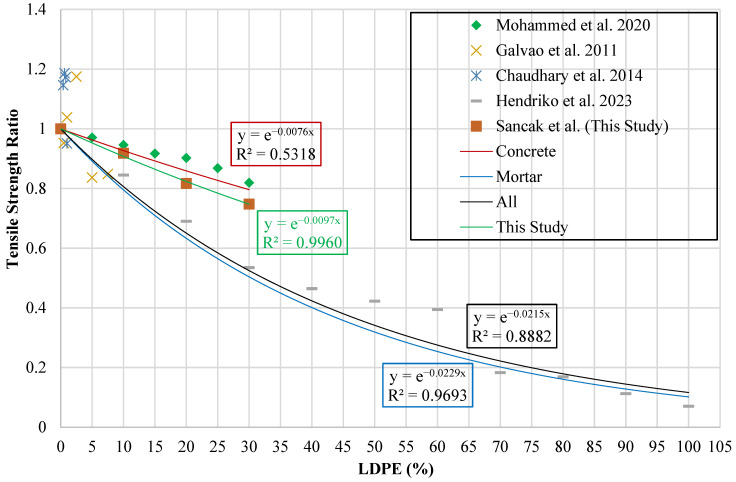
Relationship curve between tensile strength ratio and LDPE content [32,37,38,39].

A method similar to that used for generating compressive strength models was followed in generating splitting tensile strength models. A graphical representation was created where the vertical axis represents the tensile strength ratio ($\frac{f_{tldpe}^{\prime }}{f_{t}^{\prime }}$) and the horizontal axis represents the LDPE substitution percentage (ldpe%). The resulting graph is shown in Figure 10.

Trendline analyses were carried out on the plotted datasets to determine the mathematical relationship between the LDPE replacement percentage and the tensile strength ratio. The analysis considered four different groups of data: the experimental results obtained in the present study, experimental data reported in the literature for concrete mixtures, experimental data for mortar mixtures, and a combined dataset including all available results related to LDPE substitution in concrete.

The equations obtained from this analysis are given in Table 15. These equations show how the percentage of LDPE substitution affects the tensile strength. In Table 15, $f_{tldpe}^{\prime }$ is defined as the tensile strength of concrete incorporating LDPE, while $f_{t}^{\prime }$ corresponds to the tensile strength of the reference mix without LDPE. The parameter ldpe% denotes the proportion of LDPE particles employed as a substitute for fine aggregate.

Tensile strength models for LDPE-modified concrete reported in prior studies, together with the models developed in this work, were examined using statistical measures derived from the unified experimental dataset. The findings of this evaluation, along with the relevant equations, are presented in Table 16. The survey of prior studies revealed that no existing model has been published which explicitly examines the relationship between LDPE substitution levels and the tensile strength of mortar.

**Figure 11:**
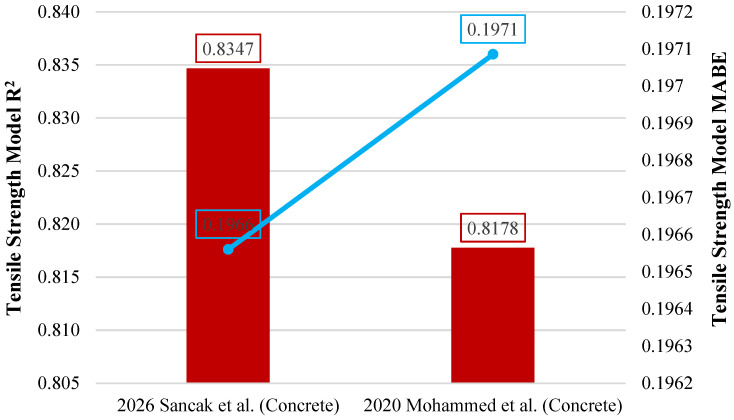
Comparison of tensile strength models for concrete based on R^2^ and MABE [32].

The tensile strength model proposed by Sancak et al. performed better than the previous model proposed by Mohammed et al. [32]. The Sancak et al. model achieved an R^2^ value of 0.835. Both approaches produced comparable predictions when applied to a limited amount of data. However, the exponential formulation proposed by Sancak et al. more consistently matched the behavior of concrete for increasing LDPE substitution levels. A comparison of the models in terms of R^2^ and MABE indicators is shown in Figure 11.

**Figure 12:**
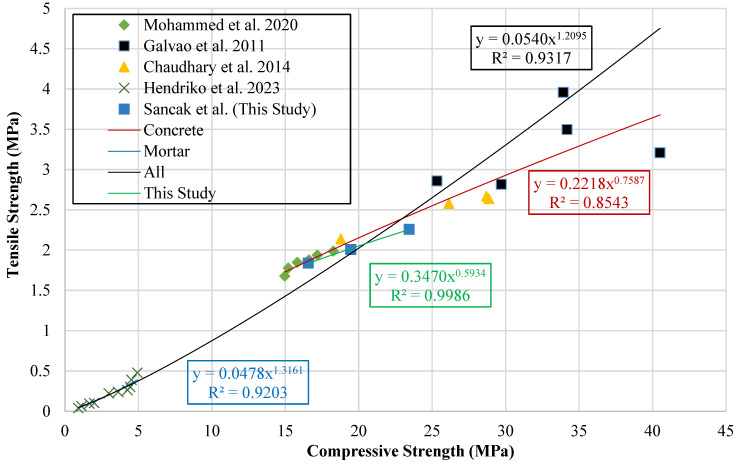
Relationship curve between tensile strength and compressive strength [32,37,38,39].

Extensive research has explored the interrelation between the compressive and tensile strengths of concrete, with several studies proposing empirical formulations to characterize this linkage. The present work critically reviews these prior approaches and seeks to establish comparable models by utilizing a consolidated experimental database. To illustrate the relationship, a graph comparing compressive and tensile strengths was prepared. During the creation of the dataset, it was observed that there were differences in sample geometry among the studies in the literature. In previous studies, compressive strength was generally measured on cubic specimens, while tensile strength was measured on cylindrical specimens. To correct for this geometric difference, cubic strength values were converted to equivalent cylindrical strengths. This comparison is shown in Figure 12.

Trendlines were used to estimate the relationship between compressive and tensile strength. These trendlines also formed the basis for the development of new prediction models. Table 17 provides the mathematical expressions obtained from the trendlines together with the associated model structures.

This study assessed previously published models describing the compressive–tensile strength relationship in LDPE concrete alongside newly formulated models, using statistical measures derived from aggregated experimental data. The findings and corresponding equations are summarized in Table 18. The literature review confirmed the absence of models addressing the compressive–tensile strength correlation in LDPE-modified mortar.

**Figure 13:**
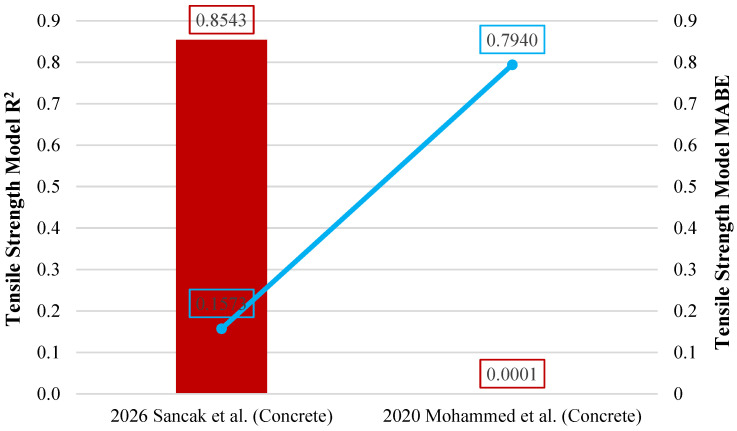
Comparison of tensile strength to compressive strength relationship models for concrete based on R^2^ and MABE [32].

The model of the relationship between tensile strength and compressive strength proposed by Sancak et al. achieved an R^2^ value of 0.854, showing a much superior performance compared to the previous model proposed by Mohammed et al. [32]. The R^2^ value of the model proposed by Mohammed et al. [32] was quite close to 0. The comparison of the models in terms of R^2^ and MABE indicators is shown in Figure 13.

### 4.3. Formulation of the Flexural Strength Prediction Model

**Figure 14:**
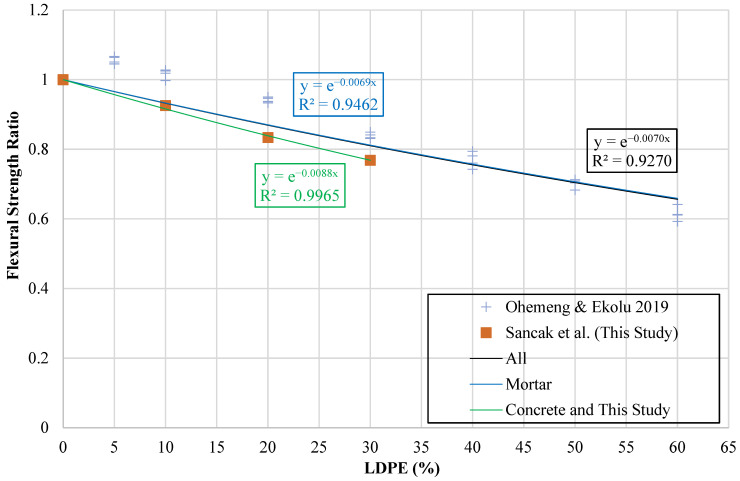
Relationship curve between flexural strength ratio and LDPE content [33].

A graph was employed to assess flexural strength, with the vertical axis expressing the ratio $\left(\frac{f_{rldpe}^{\prime }}{f_{r}^{\prime }}\right)$ and the horizontal axis showing (ldpe%). The ratio provided a normalized basis for evaluating LDPE-modified mixtures against the reference concrete. The resulting chart is displayed in Figure 14.

Trend lines were obtained expressing the mathematical relationship between the percentage of LDPE substitution and the flexural strength ratio. Separate models were proposed for concrete mixes, mortar mixes, the experimental results obtained in this study, and all results.

The mathematical expressions obtained are documented in Table 19, with $f_{rldpe}^{\prime }$ referring to the flexural strength of LDPE-substituted concrete, $f_{r}^{\prime }$ pertaining to the control mix, and ldpe% expressing the substitution ratio of LDPE particles.

**Figure 15:**
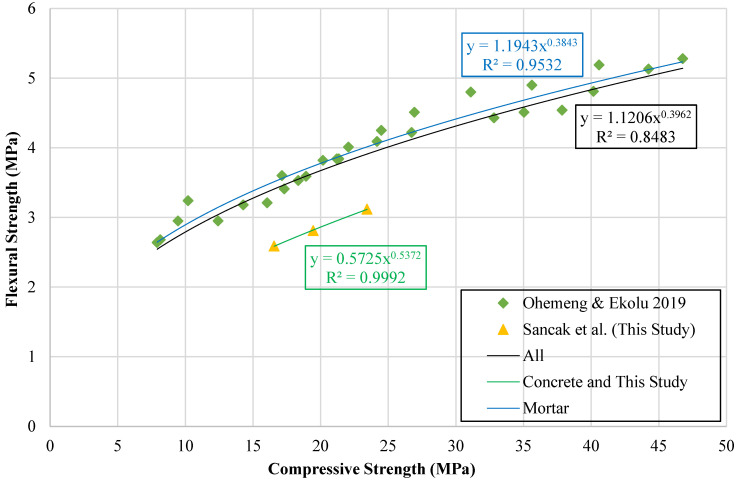
Relationship curve between flexural strength and compressive strength [33].

Extensive investigations have addressed the relationship between the compressive and flexural strengths of concrete, with several studies proposing analytical models to characterize this interaction. Building upon previously proposed models, this study creates a graph comparing compressive strength to flexural strength, as shown in Figure 15. Trend lines are plotted based on the data set in this graph. New models are then created using these trend lines to predict flexural strength from compressive strength values. The resulting equations and models are presented in Table 20.

Review of prior studies confirmed the absence of models addressing either the compressive strength–LDPE substitution ratio or the compressive–flexural strength relationship in LDPE concrete. The validity of the proposed models was examined using statistical measures, with results summarized in Table 19 and Table 20.

### 4.4. Formulation of the Modulus of Elasticity Prediction Model

**Figure 16:**
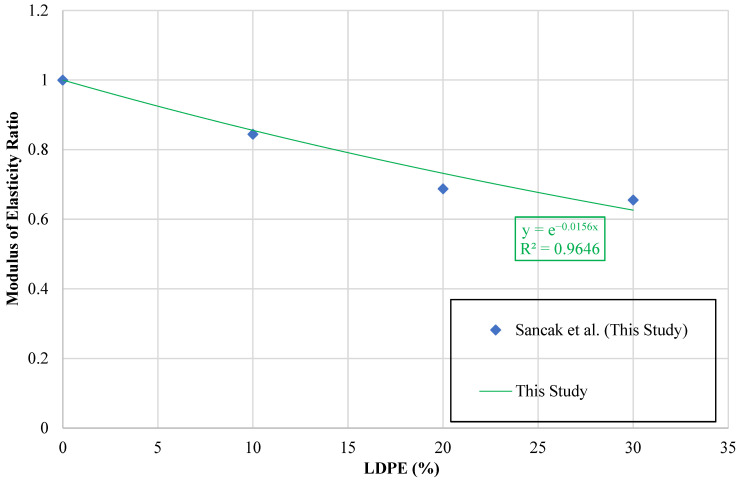
Relationship curve between modulus of elasticity ratio and LDPE content.

A plot was employed to examine the modulus of elasticity, where the vertical axis corresponds to the ratio $\left(\frac{E_{cldpe}}{E_{c}}\right)$ and the horizontal axis reflects (ldpe%). The ratio provided a normalized basis for evaluating LDPE-modified mixtures against the control concrete, with the graphical outcome presented in Figure 16.

The relationship between LDPE substitution percentage and the modulus of elasticity was modeled by applying a trendline to the observed data. The review of current literature revealed no prior studies reporting modulus of elasticity values for LDPE-modified concrete. Therefore, the fitted trendline only considers the data from this study.

The equation derived from this trendline is presented in Table 21. In Table 19, $E_{cldpe}$ is defined as the modulus of elasticity of concrete incorporating LDPE, whereas $E_{c}$ corresponds to the modulus of elasticity of the reference concrete without LDPE substitution. The parameter ldpe% designates the proportion of LDPE employed as a replacement for fine aggregate.

**Figure 17:**
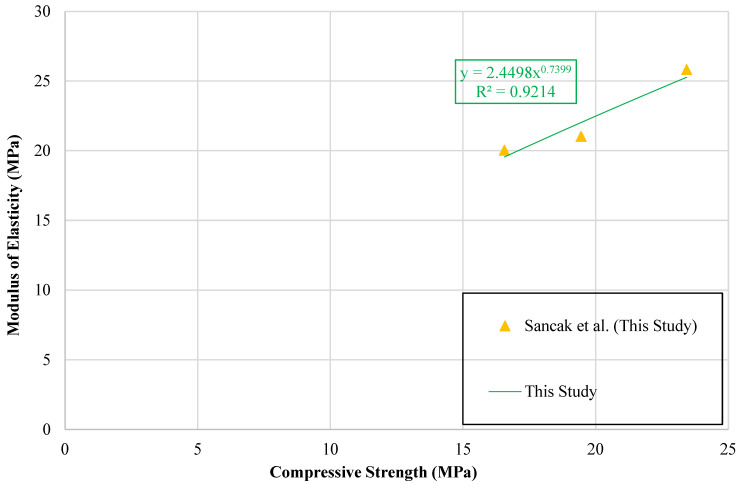
Relationship curve between modulus of elasticity and compressive strength.

A graph illustrating the relationship between compressive strength and modulus of elasticity of LDPE-substituted concrete is presented in Figure 17.

To capture the relationship between compressive strength and the modulus of elasticity, a trendline was fitted to the data, and the resulting equation is reported in Table 22.

The review of prior studies revealed a lack of models addressing compressive strength in relation to LDPE substitution or modulus of elasticity. Reliability of the models proposed in this work was examined through statistical evaluation, with results and equations presented in Table 21 and Table 22.

This study has several limitations that should be acknowledged. First, the experimental program was limited to specific LDPE substitution ratios (10%, 20%, and 30%) and a single water-to-cement ratio, which may restrict the generalizability of the results. Second, the database compiled from the literature includes variability in experimental conditions, specimen geometries, and testing standards, which may influence the accuracy of the proposed models. Although normalization procedures were applied, these differences may still introduce uncertainties. Third, the study focuses only on the mechanical properties of LDPE-modified concrete, and no microstructural analyses, such as scanning electron microscopy (SEM) or X-ray diffraction (XRD), were conducted to explain the internal mechanisms governing the observed behavior. In addition, only granular LDPE used as fine aggregate replacement was considered, excluding other forms such as fibers or hybrid plastic systems. While the increased strain capacity is documented, the practical implications of this behavior on structural performance—such as specific seismic resistance parameters or dynamic impact strength—were not directly tested in this study and remain as significant areas for future investigation. Finally, the proposed models are based on available experimental data and may require further validation with larger and more diverse datasets to ensure broader applicability.

## 5. Conclusions

This study experimentally investigated the effect of using LDPE as a partial substitute for river sand in concrete. For concrete and mortar, a dataset was created by combining the findings of the current research with data reported in previous studies. This database was used to formulate the effect of LDPE on concrete and mortar. New prediction models were developed for LDPE-substituted concrete and mortar, and the performance of existing models in the literature was evaluated. The following results were obtained:

- Substituting LDPE granules for river sand tended to reduce the workability of fresh concrete. A decrease in slump values occurred. At the 30% replacement level, there was approximately a 27% decrease.
- Increasing the LDPE content also caused a decrease in the density of the concrete. At a 30% replacement level, the density was reduced by approximately 9%. This reduction indicates that LDPE-substituted mixtures may be beneficial for the production of lightweight concrete.
- It has also been observed that the addition of LDPE reduces the modulus of elasticity, compressive strength, splitting tensile strength, and flexural strength of concrete. However, the ultimate strain values increased. These findings imply that LDPE-substituted concrete may be advantageous in situations requiring more ductility than strength.
- The proposed exponential models demonstrated higher prediction accuracy than previously suggested linear models, achieving R^2^ values of up to 0.981 and MAPE values ranging between approximately 1% and 17%. Nonetheless, further investigations are required to clarify the behavior of concrete and mortar incorporating higher levels of LDPE substitution.
- The accuracy of the developed models is inherently dependent on both the quantity and consistency of the experimental data. Variations among individual studies may influence predictive reliability. Therefore, further validation with larger and more consistent datasets is recommended.

## Figures and Tables

**Table 1:** Sieve analysis results for coarse aggregate.

Sieve Size	Remaining Weight	Passing Weight	Passing Percentage
31.5	0.000	6307.808	100.000
22.4	380.432	5927.376	93.969
16	2466.327	3461.049	54.869
11.2	2055.736	1405.313	22.279
8	609.862	795.451	12.611
5.6	301.789	493.662	7.826
4	171.132	322.530	5.113
2	127.205	195.325	3.097
1	60.175	135.150	2.143
0.500	53.621	81.529	1.293
0.250	50.543	30.986	0.491
0.125	20.121	10.865	0.172
0.063	10.865	0.000	0.000
<0.063	0	0.000	0.000

**Table 2:** Sieve analysis results for fine aggregate.

Sieve Size	Remaining Weight	Passing Weight	Passing Percentage
11.2	0.000	5052.349	100.000
8	2.696	5049.653	99.947
5.6	93.750	4955.903	98.091
4	167.410	4788.493	94.778
2	637.050	4151.444	82.169
1	1289.280	2862.164	56.650
0.500	1319.057	1543.107	30.542
0.250	920.755	622.353	12.318
0.125	601.784	20.569	0.407
0.063	20.089	0.479	0.009
<0.063	0.479	0.000	0.000

**Table 3:** LDPE granule properties and aggregate properties.

	Specific Gravity	Density (kg/m^3^)	Size (mm)
LDPE	0.92	912	2
Coarse Aggregate	2.7	1950	2–22.4
Fine Aggregate	2.6	1800	0–8

**Table 4:** Mix designs and specimen details.

Sample Name	River Sand (kg/m^3^)	LDPE (kg/m^3^)	LDPE Content Vol (%)	Water (kg/m^3^)	Cement (kg/m^3^)	Crushed Stone (kg/m^3^)	Strength Test
CS-R-0	730	-	-	195	390	1060	Compressive
CS-LDPE-10	657	36.99	10				
CS-LDPE-20	584	73.97	20				
CS-LDPE-30	511	110.96	30				
STS-R-0	730	-	-	195	390	1060	Splitting tensile
STS-LDPE-10	657	36.99	10				
STS-LDPE-20	584	73.97	20				
STS-LDPE-30	511	110.96	30				
FS-R-0	730	-	-	195	390	1060	Flexural
FS-LDPE-10	657	36.99	10				
FS-LDPE-20	584	73.97	20				
FS-LDPE-30	511	110.96	30				

**Table 5:** Summary of experimental results.

LDPE Content Vol (%)	Compressive Strength (MPa)	Splitting Tensile Strength (MPa)	Flexural Strength (MPa)	Modulus of Elasticity (MPa)	Density (kg/m^3^)	Slump (mm)
-	26.91	2.46	3.37	30,574.17	2307.88	48
10	23.43	2.26	3.12	25,815.23	2215.18	44
20	19.45	2.01	2.81	21,027.20	2175.53	40
30	16.56	1.84	2.59	20,039.28	2101.71	35

**Table 6:** Compressive strength performance of the test specimens.

Sample Name	$\mathrm{Maximum}\;\mathrm{Compressive}\;\mathrm{Stress}\;(\mathrm{MPa})\;(f_{co}^{\prime }$)	$\mathrm{Axial}\;\mathrm{Strain}\;(\varepsilon_{co}$) at Maximum Stress	$\mathrm{Lateral}\;\mathrm{Strain}\;(\varepsilon_{lo}$) at Maximum Stress
CS-R-0	26.91	0.001967	0.000747
CS-LDPE-10	23.43	0.002090	0.000820
CS-LDPE-20	19.45	0.002195	0.001032
CS-LDPE-30	16.56	0.002305	0.001111

**Table 7:** Split tensile strength performance of the test specimens.

Sample Name	Split Tensile Strength (MPa)
STS-R-0	2.46
STS-LDPE-10	2.26
STS-LDPE-20	2.01
STS-LDPE-30	1.84

**Table 8:** Flexural strength performance of the test specimens.

Sample Name	Maximum Flexural Stress (MPa)	Maximum Flexural Strain
FS-R-0	3.37	0.010467
FS-LDPE-10	3.12	0.011907
FS-LDPE-20	2.81	0.013432
FS-LDPE-30	2.59	0.014015

**Table 9:** Modulus of elasticity of the test specimens.

Sample Name	Modulus of Elasticity (MPa)
CS-R-0	30,574.17
CS-LDPE-10	25,815.23
CS-LDPE-20	21,027.20
CS-LDPE-30	20,039.28

**Table 10:** Database of experimental results for LDPE-substituted specimens.

Type of Material	Type of Substitution	Year	Reference	28-Day Compressive Strength Test (mm)	28-Day Tensile Strength Test (mm)	28-Day Flexural Strength Test (mm)	LDPE Specific Gravity	LDPE Bulk Density (kg/m^3^)	LDPE Size (mm)	LDPE (%)	Concrete Density (kg/m^3^)	W/C Ratio	28-Day Compressive Strength (MPa)	28-Day Cylindrical CS (MPa)	28-Day Tensile Strength (MPa)	28-Day Cylindrical STS (MPa)	28-Day Flexural Strength (MPa)	Modulus of Elasticity (GPa)	Slump (mm)
Concrete	Vol	2020	Mohammed et al. [32]	100 cubes	100 × 200 cylinders	—	—	—	—	0	2450	0.4	24.54	19.63	2.05	2.05	—	—	—
				100 cubes	100 × 200 cylinders	—	0.94	1440	<4.75	5	2329	0.4	22.84	18.27	1.99	1.99	—	—	—
				100 cubes	100 × 200 cylinders	—	0.94	1440	<4.75	10	2316	0.4	21.48	17.18	1.94	1.94	—	—	—
				100 cubes	100 × 200 cylinders	—	0.94	1440	<4.75	15	2304	0.4	20.76	16.61	1.88	1.88	—	—	—
				100 cubes	100 × 200 cylinders	—	0.94	1440	<4.75	20	2272	0.4	19.77	15.82	1.85	1.85	—	—	—
				100 cubes	100 × 200 cylinders	—	0.94	1440	<4.75	25	2265	0.4	19	15.20	1.78	1.78	—	—	—
				100 cubes	100 × 200 cylinders	—	0.94	1440	<4.75	30	2240	0.4	18.7	14.96	1.68	1.68	—	—	—
Concrete	Vol	2026	Sancak et al. (This Study)	100 × 200 cylinders	100 × 200 cylinders	100 × 100 × 400	—	—	—	0	2307.88	0.5	26.91	26.91	2.46	2.46	3.37	30.57	48
				100 × 200 cylinders	100 × 200 cylinders	100 × 100 × 400	0.92	912	2	10	2215.18	0.5	23.43	23.43	2.26	2.26	3.12	25.82	44
				100 × 200 cylinders	100 × 200 cylinders	100 × 100 × 400	0.92	912	2	20	2175.53	0.5	19.45	19.45	2.01	2.01	2.81	21.03	40
				100 × 200 cylinders	100 × 200 cylinders	100 × 100 × 400	0.92	912	2	30	2101.71	0.5	16.56	16.56	1.84	1.84	2.59	20.04	35
Concrete	Wei	2014	Sojobi & Owamah [2]	150 cubes	—	—	—	—	—	0	2612.12	0.59	45.04	36.03	—	—	—	—	—
				150 cubes	—	—	0.92	363.9–403.2	<2	5	2550	0.59	34.27	27.42	—	—	—	—	—
				150 cubes	—	—	0.92	363.9–403.2	<2	10	2527.27	0.59	28.66	22.93	—	—	—	—	—
				150 cubes	—	—	0.92	363.9–403.2	<2	15	2419.7	0.59	22.63	18.10	—	—	—	—	—
				150 cubes	—	—	—	—	—	0	2423.57	0.62	29.61	23.69	—	—	—	—	—
				150 cubes	—	—	0.92	363.9–403.2	<2	5	2590.11	0.62	25.34	20.27	—	—	—	—	—
				150 cubes	—	—	0.92	363.9–403.2	<2	10	2489.73	0.62	27.19	21.75	—	—	—	—	—
				150 cubes	—	—	0.92	363.9–403.2	<2	15	2389.35	0.62	20.36	16.29	—	—	—	—	—
				150 cubes	—	—	—	—	—	0	2810.1	0.6	33.75	27.00	—	—	—	—	—
				150 cubes	—	—	0.92	363.9–403.2	<2	5	2593.06	0.6	19.63	15.70	—	—	—	—	—
				150 cubes	—	—	0.92	363.9–403.2	<2	10	2580.28	0.6	21.99	17.59	—	—	—	—	—
				150 cubes	—	—	0.92	363.9–403.2	<2	15	2427.13	0.6	27.28	21.82	—	—	—	—	—
Concrete	Wei	2017	Sule et al. [36]	150 cubes	—	—	—	—	—	0	2467	0.65	20.62	16.50	—	—	—	—	78
				150 cubes	—	—	0.76	—	<5	5	2453	0.65	20.44	16.35	—	—	—	—	50
				150 cubes	—	—	0.76	—	<5	10	2216	0.65	19.69	15.75	—	—	—	—	24
				150 cubes	—	—	0.76	—	<5	15	2284	0.65	16.8	13.44	—	—	—	—	8
				150 cubes	—	—	0.76	—	<5	20	2243	0.65	15.9	12.72	—	—	—	—	3
				150 cubes	—	—	0.76	—	<5	30	2077	0.65	9.64	7.712	—	—	—	—	1
Concrete	Wei	2011	Galvao et al. [37]	NBR 5739/07	NBR 7222/94	—	—	—	—	0	—	0.45	34.35	34.35	3.37	3.37	—	—	—
				NBR 5739/07	NBR 7222/94	—	—	860	0.15–4.8	0.5	—	0.45	40.51	40.51	3.21	3.21	—	—	—
				NBR 5739/07	NBR 7222/94	—	—	860	0.15–4.8	1	—	0.45	34.18	34.18	3.5	3.5	—	—	—
				NBR 5739/07	NBR 7222/94	—	—	860	0.15–4.8	2.5	—	0.45	33.92	33.92	3.96	3.96	—	—	—
				NBR 5739/07	NBR 7222/94	—	—	860	0.15–4.8	5	—	0.45	29.7	29.7	2.82	2.82	—	—	—
				NBR 5739/07	NBR 7222/94	—	—	860	0.15–4.8	7.5	—	0.45	25.32	25.32	2.86	2.86	—	—	—
Concrete	Wei	2014	Chaudhary et al. [38]	100 cubes	150 × 150 cylinders	—	—	—	—	0	—	0.46	26.67	21.34	2.25	2.25	—	—	—
				100 cubes	150 × 150 cylinders	—	0.93	—	0.075–2.36	0.4	—	0.46	32.67	26.14	2.58	2.58	—	—	—
				100 cubes	150 × 150 cylinders	—	0.93	—	0.075–2.36	0.6	—	0.46	35.86	28.69	2.67	2.67	—	—	—
				100 cubes	150 × 150 cylinders	—	0.93	—	0.075–2.36	0.8	—	0.46	36.07	28.86	2.64	2.64	—	—	—
				100 cubes	150 × 150 cylinders	—	0.93	—	0.075–2.36	1	—	0.46	23.47	18.78	2.14	2.14	—	—	—
Concrete	Wei	2021	Olofinnade et al. [31]	100 cubes	—	—	—	—	—	0	2439.34	0.5	41.51	33.21	—	—	—	—	89.96
				100 cubes	—	—	1.11	1020	0.9–2	10	2450.37	0.5	32.04	25.63	—	—	—	—	84.90
				100 cubes	—	—	1.11	1020	0.9–2	30	2261.76	0.5	15.97	12.78	—	—	—	—	54.71
				100 cubes	—	—	1.11	1020	0.9–2	50	1948.53	0.5	12.17	9.74	—	—	—	—	14.81
Mortar	Vol	2019	Ohemeng & Ekolu [33]	50 cubes	—	40 × 40 × 160	—	—	—	0	2290	0.45	55.38	44.30	—	—	5.05	—	—
				50 cubes	—	40 × 40 × 160	1.1	813.6	<4.75	5	2189	0.45	46.76	37.41	—	—	5.28	—	—
				50 cubes	—	40 × 40 × 160	1.1	813.6	<4.75	10	2154	0.45	40.58	32.46	—	—	5.19	—	—
				50 cubes	—	40 × 40 × 160	1.1	813.6	<4.75	20	2066	0.45	31.1	24.88	—	—	4.8	—	—
				50 cubes	—	40 × 40 × 160	1.1	813.6	<4.75	30	1930	0.45	24.49	19.59	—	—	4.25	—	—
				50 cubes	—	40 × 40 × 160	1.1	813.6	<4.75	40	1850	0.45	21.34	17.07	—	—	3.84	—	—
				50 cubes	—	40 × 40 × 160	1.1	813.6	<4.75	50	1722	0.45	17.14	13.71	—	—	3.6	—	—
				50 cubes	—	40 × 40 × 160	1.1	813.6	<4.75	60	1701	0.45	10.2	8.16	—	—	3.24	—	—
				50 cubes	—	40 × 40 × 160	—	—	—	0	2274	0.5	52.17	41.74	—	—	4.81	—	—
				50 cubes	—	40 × 40 × 160	1.1	813.6	<4.75	5	2174	0.5	44.24	35.39	—	—	5.13	—	—
				50 cubes	—	40 × 40 × 160	1.1	813.6	<4.75	10	2141	0.5	35.61	28.49	—	—	4.9	—	—
				50 cubes	—	40 × 40 × 160	1.1	813.6	<4.75	20	2018	0.5	26.93	21.54	—	—	4.51	—	—
				50 cubes	—	40 × 40 × 160	1.1	813.6	<4.75	30	1903	0.5	22.06	17.65	—	—	4.01	—	—
				50 cubes	—	40 × 40 × 160	1.1	813.6	<4.75	40	1839	0.5	20.17	16.14	—	—	3.82	—	—
				50 cubes	—	40 × 40 × 160	1.1	813.6	<4.75	50	1709	0.5	17.31	13.85	—	—	3.41	—	—
				50 cubes	—	40 × 40 × 160	1.1	813.6	<4.75	60	1690	0.5	9.46	7.57	—	—	2.95	—	—
				50 cubes	—	40 × 40 × 160	—	—	—	0	2231	0.55	48.21	38.57	—	—	4.52	—	—
				50 cubes	—	40 × 40 × 160	1.1	813.6	<4.75	5	2160	0.55	40.16	32.13	—	—	4.81	—	—
				50 cubes	—	40 × 40 × 160	1.1	813.6	<4.75	10	2132	0.55	35.03	28.02	—	—	4.51	—	—
				50 cubes	—	40 × 40 × 160	1.1	813.6	<4.75	20	2009	0.55	26.73	21.38	—	—	4.22	—	—
				50 cubes	—	40 × 40 × 160	1.1	813.6	<4.75	30	1891	0.55	21.19	16.95	—	—	3.84	—	—
				50 cubes	—	40 × 40 × 160	1.1	813.6	<4.75	40	1821	0.55	18.35	14.68	—	—	3.53	—	—
				50 cubes	—	40 × 40 × 160	1.1	813.6	<4.75	50	1695	0.55	14.28	11.42	—	—	3.18	—	—
				50 cubes	—	40 × 40 × 160	1.1	813.6	<4.75	60	1672	0.55	8.15	6.52	—	—	2.68	—	—
				50 cubes	—	40 × 40 × 160	—	—	—	0	2216	0.6	45.41	36.33	—	—	4.32	—	—
				50 cubes	—	40 × 40 × 160	1.1	813.6	<4.75	5	2149	0.6	37.85	30.28	—	—	4.54	—	—
				50 cubes	—	40 × 40 × 160	1.1	813.6	<4.75	10	2117	0.6	32.82	26.26	—	—	4.43	—	—
				50 cubes	—	40 × 40 × 160	1.1	813.6	<4.75	20	1998	0.6	24.17	19.34	—	—	4.09	—	—
				50 cubes	—	40 × 40 × 160	1.1	813.6	<4.75	30	1872	0.6	18.92	15.14	—	—	3.59	—	—
				50 cubes	—	40 × 40 × 160	1.1	813.6	<4.75	40	1801	0.6	16.05	12.84	—	—	3.21	—	—
				50 cubes	—	40 × 40 × 160	1.1	813.6	<4.75	50	1679	0.6	12.42	9.94	—	—	2.95	—	—
				50 cubes	—	40 × 40 × 160	1.1	813.6	<4.75	60	1655	0.6	7.91	6.33	—	—	2.64	—	—
Mortar	Vol	2020	Mohammed et al. [32]	50 cubes	—	—	—	—	—	0	2387	0.4	42.79	34.23	—	—	—	—	—
				50 cubes	—	—	0.94	1440	<4.75	5	2176	0.4	29.02	23.22	—	—	—	—	—
				50 cubes	—	—	0.94	1440	<4.75	10	2147	0.4	27.72	22.18	—	—	—	—	—
				50 cubes	—	—	0.94	1440	<4.75	15	2141	0.4	26.41	21.13	—	—	—	—	—
				50 cubes	—	—	0.94	1440	<4.75	20	2112	0.4	25.37	20.30	—	—	—	—	—
				50 cubes	—	—	0.94	1440	<4.75	25	2101	0.4	24.61	19.69	—	—	—	—	—
Mortar	Wei	2024	Hendriko et al. [39]	50 cubes	50 cubes	—	—	—	—	0	1600	0.57	10.49	8.39	0.71	0.57	—	—	—
				50 cubes	50 cubes	—	—	938	0.6	10	1442.67	0.71	6.15	4.92	0.60	0.48	—	—	—
				50 cubes	50 cubes	—	—	938	0.6	20	1296	0.86	5.66	4.53	0.49	0.39	—	—	—
				50 cubes	50 cubes	—	—	938	0.6	30	1264	1	5.55	4.44	0.38	0.30	—	—	—
				50 cubes	50 cubes	—	—	938	0.6	40	1184	1.14	5.33	4.26	0.33	0.26	—	—	—
				50 cubes	50 cubes	—	—	938	0.6	50	1045.33	1.29	4.54	3.63	0.30	0.24	—	—	—
				50 cubes	50 cubes	—	—	938	0.6	60	917.33	1.43	3.73	2.98	0.28	0.22	—	—	—
				50 cubes	50 cubes	—	—	938	0.6	70	805.33	1.71	2.49	1.99	0.13	0.10	—	—	—
				50 cubes	50 cubes	—	—	938	0.6	80	714.67	2	2.09	1.67	0.12	0.09	—	—	—
				50 cubes	50 cubes	—	—	938	0.6	90	666.67	2.29	1.41	1.13	0.08	0.06	—	—	—
				50 cubes	50 cubes	—	—	938	0.6	100	613.33	2.57	1.14	0.91	0.05	0.04	—	—	—

**Table 11:** Statistical evaluation indicators.

Metric	Equation
R^2^	${\left(\frac{\sum \left({exp}_{i}-{\bar{exp}}_{i}\right)\left({mod}_{i}-{\bar{mod}}_{i}\right)}{\sqrt{\sum {\left({exp}_{i}-{\bar{exp}}_{i}\right)}^{2}\sum {\left({mod}_{i}-{\bar{mod}}_{i}\right)}^{2}}}\right)}^{2}$
MABE	$\frac{1}{n}\sum_{i=1}^{n}\left|{mod}_{i}-{exp}_{i}\right|$
MAPE	$\frac{1}{n}\sum_{i=1}^{n}\left|\frac{{exp}_{i}-{mod}_{i}}{{exp}_{i}}\right|\times 100$

**Table 12:** Established compressive strength functions and their associated models.

Type of Data	Proposed Model	Data Point	R^2^	MABE	MAPE
Experimental Data of This Study	$f_{cldpe}^{\prime }=\left(f_{c}^{\prime }\right)\left(e^{-0.0160(ldpe\%)}\right)$	3	0.998	0.227	1.049
Concrete Data	$f_{cldpe}^{\prime }=\left(f_{c}^{\prime }\right)\left(e^{-0.0207(ldpe\%)}\right)$	35	0.719	3.213	16.629
Mortar Data	$f_{cldpe}^{\prime }=\left(f_{c}^{\prime }\right)\left(e^{-0.0238(ldpe\%)}\right)$	43	0.981	1.845	15.042
All Data	$f_{cldpe}^{\prime }=\left(f_{c}^{\prime }\right)\left(e^{-0.0234(ldpe\%)}\right)$	78	0.894	2.516	15.420

**Table 13:** Performance assessment of the proposed compressive strength models for concrete.

Year	Reference	Data Point	R^2^	MABE	MAPE
2020	Mohammed et al. [32]				
	$f_{cldpe}^{\prime }=0.9738-0.0078(ldpe\%)$	35	0.549	3.858	114.720
2026	Sancak et al. (this study)				
	$f_{cldpe}^{\prime }=\left(f_{c}^{\prime }\right)\left(e^{-0.0207(ldpe\%)}\right)$	35	0.719	3.213	16.629

**Table 14:** Performance assessment of the proposed compressive strength models for mortar.

Year	Reference	Data Point	R^2^	MABE	MAPE
2019	Ohemeng and Ekolu [33] (Mortar I)				
	$f_{cldpe}^{\prime }=0.0689\gamma_{ldpe}-106.17$	43	0.759	16.256	572.510
2019	Ohemeng and Ekolu [33] (Mortar II)				
	$f_{cldpe}^{\prime }=-34.306w/c-0.529P+0.459T+47.775$	43	0.576	14.127	616.208
2020	Mohammed et al. [32]				
	$f_{cldpe}^{\prime }=0.9494-0.0414(ldpe\%)+0.0011{\left(ldpe\%\right)}^{2}$	43	0.069	22.624	656.311
2025	Sancak et al. (this study)				
	$f_{cldpe}^{\prime }=\left(f_{c}^{\prime }\right)\left(e^{-0.0238(ldpe\%)}\right)$	43	0.981	1.845	15.042

**Table 15:** Established tensile strength functions and their associated models.

Type of Data	Proposed Model	Data Point	R^2^	MABE	MAPE
Experimental Data of This Study	$f_{tldpe}^{\prime }=\left(f_{t}^{\prime }\right)\left(e^{-0.0097(ldpe\%)}\right)$	3	0.993	0.015	0.693
Concrete Data	$f_{tldpe}^{\prime }=\left(f_{t}^{\prime }\right)\left(e^{-0.0076(ldpe\%)}\right)$	18	0.835	0.197	7.287
Mortar Data	$f_{tldpe}^{\prime }=\left(f_{t}^{\prime }\right)\left(e^{-0.0229(ldpe\%)}\right)$	10	0.959	0.030	16.667
All Data	$f_{tldpe}^{\prime }=\left(f_{t}^{\prime }\right)\left(e^{-0.0215(ldpe\%)}\right)$	28	0.955	0.237	17.137

**Table 16:** Performance assessment of the proposed tensile strength models for concrete.

Year	Reference	Data Point	R^2^	MABE	MAPE
2020	Mohammed et al. [32]				
	$f_{tldpe}^{\prime }=1.0007-0.0056(ldpe\%)$	18	0.818	0.197	7.289
2026	Sancak et al. (this study)				
	$f_{tldpe}^{\prime }=\left(f_{t}^{\prime }\right)\left(e^{-0.0076(ldpe\%)}\right)$	18	0.835	0.197	7.287

**Table 17:** Equations and models relating tensile strength to compressive strength.

Type of Data	Proposed Model	Data Point	R^2^	MABE	MAPE
Experimental Data of This Study	$f_{tldpe}^{\prime }=0.3470{\left(f_{cldpe}^{\prime }\right)}^{0.5934}$	3	0.999	0.006	0.310
Concrete Data	$f_{tldpe}^{\prime }=0.2218{\left(f_{cldpe}^{\prime }\right)}^{0.7587}$	18	0.854	0.157	5.426
Mortar Data	$f_{tldpe}^{\prime }=0.0478{\left(f_{cldpe}^{\prime }\right)}^{1.3161}$	10	0.920	0.030	11.689
All Data	$f_{tldpe}^{\prime }=0.0540{\left(f_{cldpe}^{\prime }\right)}^{1.2095}$	28	0.932	0.232	13.534

**Table 18:** Performance evaluation of proposed tensile strength to compressive strength relationship models for concrete.

Year	Reference	Data Point	R^2^	MABE	MAPE
2020	Mohammed et al. [32]				
	$f_{tldpe}^{\prime }=-2.6341+0.3679{(f}_{cldpe}^{\prime })-0.0072{\left(f_{cldpe}^{\prime }\right)}^{2}$	18	0.0001	0.794	29.101
2026	Sancak et al. (this study)				
	$f_{tldpe}^{\prime }=0.2218{\left(f_{cldpe}^{\prime }\right)}^{0.7587}$	18	0.854	0.157	5.426

**Table 19:** Established flexural strength functions and their associated models.

Type of Data	Proposed Model	Data Point	R^2^	MABE	MAPE
Experimental Data of This Study and Concrete Data	$f_{rldpe}^{\prime }=\left(f_{r}^{\prime }\right)\left(e^{-0.0088(ldpe\%)}\right)$	3	0.995	0.017	0.578
Mortar Data	$f_{rldpe}^{\prime }=\left(f_{r}^{\prime }\right)\left(e^{-0.0069(ldpe\%)}\right)$	28	0.986	0.230	5.536
All Data	$f_{rldpe}^{\prime }=\left(f_{r}^{\prime }\right)\left(e^{-0.0070(ldpe\%)}\right)$	31	0.989	0.219	5.356

**Table 20:** Equations and models relating flexural strength to compressive strength.

Type of Data	Proposed Model	Data Point	R^2^	MABE	MAPE
Experimental Data of This Study and Concrete Data	$f_{rldpe}^{\prime }=0.5725{\left(f_{cldpe}^{\prime }\right)}^{0.5372}$	3	0.999	0.006	0.204
Mortar Data	$f_{rldpe}^{\prime }=1.1943{\left(f_{cldpe}^{\prime }\right)}^{0.3843}$	28	0.953	0.330	8.131
All Data	$f_{rldpe}^{\prime }=1.1206{\left(f_{cldpe}^{\prime }\right)}^{0.3962}$	31	0.747	0.469	12.553

**Table 21:** Established modulus of elasticity function and its associated model.

Type of Data	Proposed Model	Data Point	R^2^	MABE	MAPE
Experimental Data of This Study	$E_{cldpe}=\left(E_{c}\right)\left(e^{-0.0156(ldpe\%)}\right)$	3	0.902	0.859	4.056

**Table 22:** Equation and model relating modulus of elasticity to compressive strength.

Type of Data	Proposed Model	Data Point	R^2^	MABE	MAPE
Experimental Data of This Study	$E_{cldpe}=2.4498{\left(f_{cldpe}^{\prime }\right)}^{0.7399}$	3	0.921	0.677	3.094

## Data Availability

The original contributions presented in this study are included in the article. Further inquiries can be directed to the corresponding author.

## References

[1] Ohemeng E.A., Yalley P.P., Dadzie J., Djokoto S.D. (2014). Utilization of waste low density polyethylene in high strengths concrete pavement blocks production. Civ. Environ. Res..

[2] Sojobi A.O., Owamah H.I. (2014). Evaluation of the suitability of low-density polyethylene (LDPE) waste as fine aggregate in concrete. Niger. J. Technol..

[3] Merbouh M.H., Glaoui B. (2015). Plastic Waste in Cement Concrete, LDPE and PVC introduction effect. Adv. Mater. Res..

[4] Gu L., Ozbakkaloglu T. (2016). Use of recycled plastics in concrete: A critical review. Waste Manag..

[5] Saraswat P., Singh B. (2024). Utilization of recycled concrete aggregates in LDPE-bonded cementless paver blocks. Constr. Build. Mater..

[6] Al-Tarbi S.M., Al-Amoudi O.S.B., Al-Osta M.A., Al-Awsh W.A., Ali M.R., Maslehuddin M. (2022). Development of eco-friendly hollow concrete blocks in the field using wasted high-density polyethylene, low-density polyethylene, and crumb tire rubber. J. Mater. Res. Technol..

[7] Othman A.M. (2010). Effect of low-density polyethylene on fracture toughness of asphalt concrete mixtures. J. Mater. Civ. Eng..

[8] Nursyamsi N., Indrawan I., Ramadhan P. (2019). The influence of the usage of ldpe plastic waste as fine aggregate in light concrete bricks. MATEC Web of Conferences.

[9] Rodrigues C., Capitão S., Picado-Santos L., Almeida A. (2020). Full recycling of asphalt concrete with waste cooking oil as rejuvenator and LDPE from urban waste as binder modifier. Sustainability.

[10] Sukmawan W., Arifin S. (2021). Effect of Adding Plastic Waste LDPE (Low Density Polyethylene) and PET (Polyethylene Terephthalate) on the Behaviour of Stability Marshall Characteristics of Asphalt Concrete Mixture. Int. J. Civ. Eng..

[11] Singh V., SinghRana A. (2025). Enhancing Sustainability in Concrete Construction: Utilizing LDPE and HDPE Plastics as Fine Aggregate Replacements. Rock Soil Mech..

[12] Karthik M., Ajey Kumar V.G., Keshava M. (2021). Study on behavior of concrete mixes using waste plastics as an alternative for coarse aggregates. IOP Conference Series: Earth and Environmental Science.

[13] Bahoria B.V., Parbat D.K., Nagarnaik P.B. (2018). XRD analysis of natural sand, quarry dust, waste plastic (ldpe) to be used as a fine aggregate in concrete. Mater. Today Proc..

[14] İpek S., Diri A., Mermerdaş K. (2021). Recycling the low-density polyethylene pellets in the pervious concrete production. J. Mater. Cycles Waste Manag..

[15] Mashaan N.S., Ouano C.A.E. (2025). An investigation of the mechanical properties of concrete with different types of waste plastics for rigid pavements. Appl. Mech..

[16] Mohammed H., Giuntini F., Sadique M., Shaw A., Bras A. (2022). Polymer modified concrete impact on the durability of infrastructure exposed to chloride environments. Constr. Build. Mater..

[17] Jnr A.K.L., Yunana D., Kamsouloum P., Webster M., Wilson D.C., Cheeseman C. (2018). Recycling waste plastics in developing countries: Use of low-density polyethylene water sachets to form plastic bonded sand blocks. Waste Manag..

[18] Rumšys D., Bačinskas D., Spudulis E., Meškėnas A. (2017). Comparison of material properties of lightweight concrete with recycled polyethylene and expanded clay aggregates. Procedia Eng..

[19] Hamah Sor N., Ali T.K.M., Vali K.S., Ahmed H.U., Faraj R.H., Bheel N., Mosavi A. (2022). The behavior of sustainable self-compacting concrete reinforced with low-density waste Polyethylene fiber. Mater. Res. Express.

[20] Laria J.G., Gaggino R., Kreiker J., Peisino L.E., Positieri M., Cappelletti A. (2023). Mechanical and processing properties of recycled PET and LDPE-HDPE composite materials for building components. J. Thermoplast. Compos. Mater..

[21] Poonyakan A., Rachakornkij M., Wecharatana M., Smittakorn W. (2018). Potential use of plastic wastes for low thermal conductivity concrete. Materials.

[22] Celauro C., Bosurgi G., Sollazzo G., Ranieri M. (2019). Laboratory and in-situ tests for estimating improvements in asphalt concrete with the addition of an LDPE and EVA polymeric compound. Constr. Build. Mater..

[23] Du X., Liu S., Lin H., Xu X., Zheng Z., Zhang H. (2023). Study on preparation technology and performance of polyethylene plastic concrete for road. Constr. Build. Mater..

[24] Prahara E., Aswita F., Niluh Putu Shinta E.S. (2020). The effect of High-Density Polyethylene (HDPE) and Low-Density Polyethylene (LDPE) on characteristics of asphalt concrete with dry and wet mixing process. IOP Conference Series: Materials Science and Engineering.

[25] Setyarini N.L., Tajudin A. (2019). Characteristics of asphalt concrete mixed using aggregates coated by low density polyethilene (LDPE) plastic waste. 11th Asia Pacific Transportation and the Environment Conference.

[26] Sharma A., Singh S. (2019). Experimental study on use of waste hdpe, ldpe and chloroprene rubber in bituminous concrete. Int. J. Innov. Technol. Explor. Eng..

[27] Yaghoubi E., Arulrajah A., Wong Y.C., Horpibulsuk S. (2017). Stiffness properties of recycled concrete aggregate with polyethylene plastic granules in unbound pavement applications. J. Mater. Civ. Eng..

[28] Ullah S., Raheel M., Khan R., Khan M.T. (2021). Characterization of physical & mechanical properties of asphalt concrete containing low-& high-density polyethylene waste as aggregates. Constr. Build. Mater..

[29] Zakaria R.F., Al Jauhari Z. (2023). The effect of pet and LDPE plastic wastes on the compressive strength of paving blocks. GEOMATE J..

[30] Noriya P., Dwivedi P., Scholar P. (2021). Experimental investigation of concrete utilizing plastic waste HDPE and LDPE as a replacement of aggregate in concrete a review. Int. J. Sci. Res. Civ. Eng..

[31] Olofinnade O., Chandra S., Chakraborty P. (2021). Recycling of high impact polystyrene and low-density polyethylene plastic wastes in lightweight based concrete for sustainable construction. Mater. Today Proc..

[32] Mohammed A., Ali T.K.M., Rajab N., Hilal N. (2020). Mechanical properties of concrete and mortar containing low density polyethylene waste particles as fine aggregate. J. Mater. Eng. Struct..

[33] Ohemeng E.A., Ekolu S.O. (2019). Strength prediction model for cement mortar made with waste LDPE plastic as fine aggregate. J. Sustain. Cem.-Based Mater..

[34] (2012). Tests for Geometrical Properties of Aggregates-Part 1: Determination of Particle Size Distribution-Sieving Method.

[35] (2011). Cement-Part 1: Composition, Specifications and Conformity Criteria for Common Cements.

[36] Sule J., Emmanuel S., Joseph I., Ibhadobe O., Alfred B.Y., Waziri F.I., Sunny E. (2017). Use of waste plastics in cement-based composite for lightweight concrete production. Int. J. Res. Eng. Technol..

[37] Galvão J.C.A., Portella K.F., Joukoski A., Mendes R., Ferreira E.S. (2011). Use of waste polymers in concrete for repair of dam hydraulic surfaces. Constr. Build. Mater..

[38] Chaudhary M., Srivastava V., Agarwal V. (2014). Effect of waste low density polyethylene on mechanical properties of concrete. J. Acad. Ind. Res..

[39] Hendriko A., Juwono A.L., Budiman I., Subyakto, Soegijono B., Sadir M., Sudarmanto, Purnomo D., Narto, Akbar F. (2024). Mechanical and thermal properties of non-structural adhesive mortar using linear low-density polyethylene (LLDPE) aggregate substitution with vinyl acetate/ethylene (VAE) interface. Colloid Polym. Sci..

[40] Sancak O.F., Ozyurt M.Z. (2024). PET Granule Replacement for Fine Aggregate in Concrete and FRP-Wrapping Effect: Overview of Experimental Data and Model Development. Buildings.

[41] Sancak O.F., Ozyurt M.Z. (2025). Mechanical Behavior and Predictive Modeling of Cementitious Composites Incorporating Recycled HDPE. Polymers.

[42] Eren B., Yaqub M., Eyüpoğlu V. (2016). Assessment of neural network training algorithms for the prediction of polymeric inclusion membranes efficiency. Sak. Univ. J. Sci..

[43] Lin L., Xu N., Yang D., Li G., Xiao Y., Yu Y. (2026). A smart computational framework for predicting mechanical and sustainability indicators and optimizing mix proportions of recycled rubber aggregate concrete. Eng. Appl. Artif. Intell..

